# Safety and efficacy of combined B-cell depleting therapy and daratumumab in patients with autoimmune encephalitis (RADIA): study protocol for a multicenter, randomized trial

**DOI:** 10.3389/fneur.2026.1783651

**Published:** 2026-05-29

**Authors:** Wei Xie, Lin-Jie Zhang, Chunxian Yue, Baojie Wang, Shougang Guo, Xuegan Lian, Chao Zhang

**Affiliations:** 1 Department of Neurology, The Third Affiliated Hospital of Soochow University, Changzhou, Jiangsu, China; 2 Department of Neurology, Tianjin Medical University General Hospital, Tianjin, China; 3 Department of Neurology, Shandong Provincial Hospital, Shandong First Medical University, Jinan, Shandong, China; 4 Department of Neurology, China National Clinical Research Center for Neurological Diseases, Beijing Tiantan Hospital, Capital Medical University, Beijing, China

**Keywords:** autoimmune encephalitis, daratumumab, mRS, NMDA receptors, plasma cells

## Abstract

**Background:**

Anti-NMDA receptor encephalitis (NMDARE) is the most common autoimmune encephalitis, involving an immune response against NMDA receptors on neurons. Despite standard immunotherapy, approximately 30% of critically ill patients require long-term intensive care. Current management strategies are mainly based on retrospective studies and expert opinions, with limited high-quality evidence. This study aims to evaluate the efficacy and safety of B-cell depleting agents followed by daratumumab as a second-line treatment for severe autoimmune encephalitis (AE) with NMDARE as the primary target population.

**Objective:**

To describe the study protocol for the RADIA study.

**Study design:**

This multicenter, open-label, randomized controlled trial is conducted across 58 tertiary hospitals in China. Eligible participants are patients with severe AE especially NMDARE (mRS ≥ 3 points and neuropsychiatric manifestations inadequate to symptomatic treatment) post first-line immunotherapy. A total of 200 patients will be randomized at a 2:2:1 ratio into three groups. 80 patients will receive B-cell depleting agent (anti-CD20 mAb ofatumumab) followed by anti-CD38 mAb daratumumab, 80 patients will receive B-cell depleting agent ofatumumab alone, and 40 receive repeated IVIG or plasmapheresis.

**Main outcome measures:**

The primary endpoint is the proportion of patients with an mRS score ≤ 2, assessed at 16 weeks after treatment initiation. The secondary outcomes include scores on the Clinical Assessment Scale for Autoimmune Encephalitis (CASE), neurocognitive function, antibody status, ICU stay, and hospitalization duration up to 48 weeks. Adverse events will be monitored continuously.

**Conclusion:**

This trial aims to provide robust data on the efficacy of B cell depleting agents followed by daratumumab in patients with severe AE, with a primary focus on the NMDARE subgroup. This combined regimen may provide a new treatment option for NMDARE.

**Clinical trial registration:**

https://clinicaltrials.gov/study/NCT06867991, identifier (NCT06867991).

## 1 Introduction

Autoimmune encephalitis (AE) is a spectrum of inflammatory disorders in the central nervous system, usually driven by aberrant immune responses targeting neural antigens including surface antigens and intracellular antigens (1). The underlying immunopathology in individuals with neural surface autoantiboides is believed to be antibody mediated, irrespective of the presence of neoplasms. Though AE is a rare heterogenous disease, it can affect patients of all ages, both in children and young adults (2). Anti-N-methyl-D-aspartate receptor encephalitis (NMDARE) is the most common AE, followed by leucine-rich glioma-inactivated 1 (LGI1), gamma-aminobutyric acid type B receptor (GABABR), contactin-associated protein-like 2 (CASPR2) and other encephalitis (3). AE usually presents with mental and behavioral changes, seizures, movement disorders, and autonomic dysfunction. Severe cases may have impaired consciousness, status epilepticus, status dystonia, autonomic instability, and central hypoventilation requiring mechanical ventilation (4). Current treatment regimens often adopt a stepwise approach. First-line immunotherapies include intravenous methylprednisolone (IVMP), intravenous immunoglobulin (IVIG), and plasma exchange (PE) or immunoadsorption. For patients who show inadequate responses to initial treatments, second-line therapies are used, including B-cell depleting therapy (BCDT) or cyclophosphamide (5, 6). Rituximab is the most frequently used anti-CD20 monoclonal antibody (mAb) in AE.

Real-world evidence from the GENERATE registry has demonstrated that rituximab, the most frequently used anti-CD20 monoclonal antibody in AE, is associated with improved functional outcomes in NMDAR, LGI1, and CASPR2 encephalitis and a reduced relapse rate, although limited benefit is observed in long-standing GAD65 disease (7). Recent studies showed that another mAb, ofatumumab, which was approved in multiple sclerosis, may also have a beneficial effect on AE. More recently, the prospective OFF-AE cohort study reported that ofatumumab, a second-generation anti-CD20 antibody, led to further functional improvement in 93.1% of NMDAR encephalitis patients, with a particularly favorable effect in those who failed first-line immunotherapy (87.9% vs. 64.7% achieving mRS ≤ 2) and no serious adverse events (8). However, a recent real-world prospective cohort study involving severe anti-NMDAR encephalitis found that only 63.9% of ofatumumab-treated and 55.6% of rituximab-treated patients achieved a good outcome [modified Rankin Scale (mRS) ≤ 2] at 3 months, although BCDT significantly improved outcomes compared to conventional first-line immunotherapy alone (9). Another report showed that approximately 30% of critically ill patients do not respond to these existing treatments and require long-term intensive care support (10). Therefore, there are still urgent unmet needs for severe patients with AE to achieve a good outcome.

Plasma cells reside within the central nervous system and its borders, where they contribute to both protective humoral immunity and pathological inflammation (11). Pathological studies found a significant infiltration of antibody-secreting cells (CD38^+^ CD138^+^) into the brain tissue of AE patients (12, 13). These cells are found around blood vessels, within interstitial spaces, and in Virchow-Robin spaces, where they continue to secrete autoantibodies, thus explaining the synthesis of intrathecal antibodies. Traditional B-cell depleting agents, such as rituximab may deplete short-lived plasma cells and plasmablasts derived from activated B cells but not CD20-negative long-lived plasma cells (14), which may contribute to treatment failure in severe NMDARE cases. Given these pathological insights, plasma cell-targeted therapy has emerged as a promising area of research (15). Daratumumab, an anti-CD38 monoclonal antibody, exerts its therapeutic effects by specifically eliminating short-lived and long-lived plasma cells and plasmablasts that express high levels of CD38. The drug has demonstrated high efficacy in multiple myeloma (16) and exhibits immunomodulatory functions on some other cells, including effector memory CD8^+^ T cells (17). Recent case reports indicate that daratumumab has significant potential for the treatment of refractory AE (18, 19). In parallel, the ongoing phase 2B EXTINGUISH trial is evaluating inebilizumab, an anti-CD19 antibody that depletes a broader spectrum of B-lineage cells including plasmablasts and some plasma cells, in a randomized, placebo-controlled design involving 116 patients with moderate-to-severe NMDAR encephalitis, with primary outcome assessed at 16 weeks (20). It remains to be determined whether early use of plasma cell depleting treatment provide more benefits in patients with severe AE. This trial aims to investigate the efficacy of daratumumab add-on ofatumumab in patients with severe AE and autoantibodies against neural surface antigens.

## 2 Methods

### 2.1 Trial design and setting

RADIA is a randomized, controlled, open-label, multicenter clinical trial initiated by investigators to assess the safety and efficacy of B-cell depleting therapy followed by daratumumab in patients with severe AE, with NMDARE as the primary target population. The study will be conducted in the Department of Neurology in 58 tertiary hospitals nationwide, with a planned enrollment of 200 patients by December 2026.

**Figure 1:**
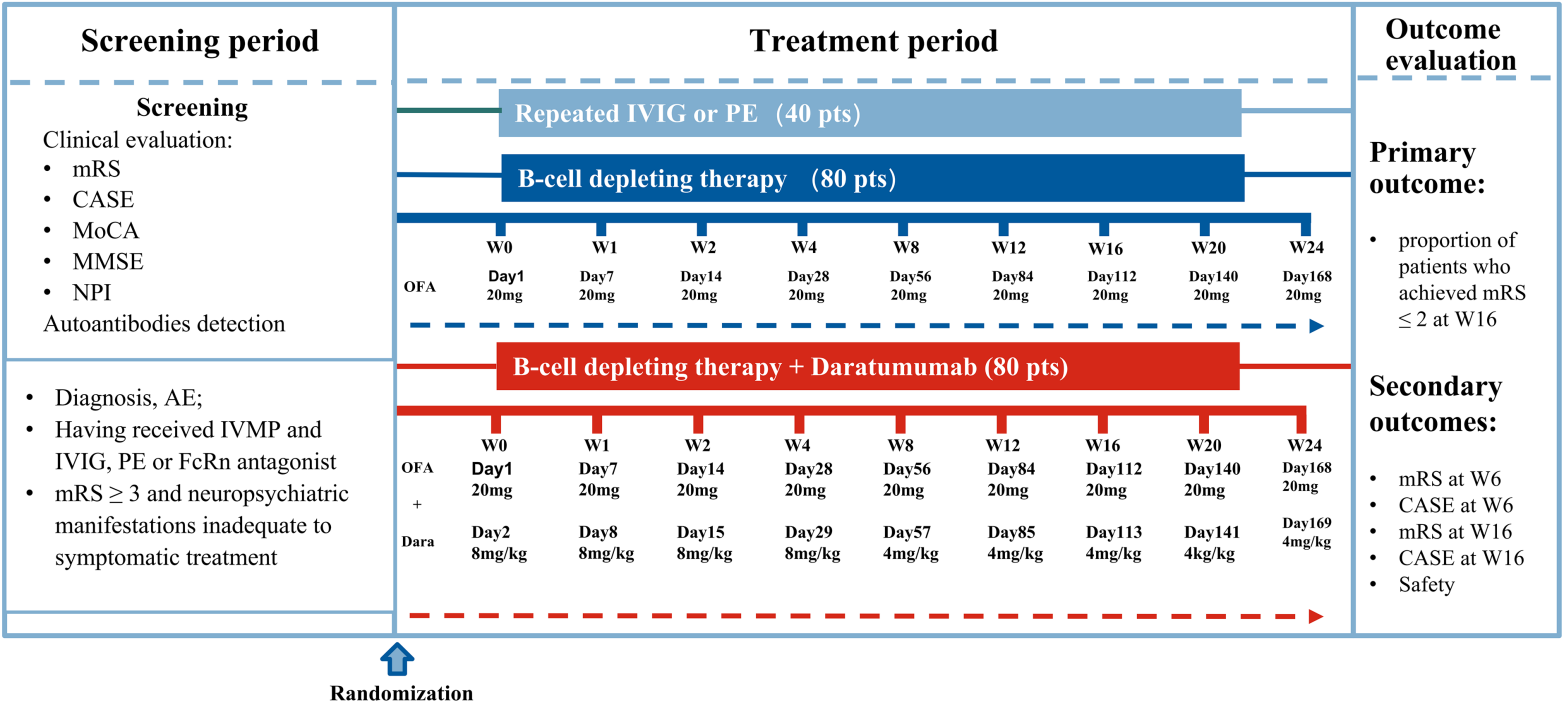
Outline of the study procedure. OFA, ofatumumab; Dara, daratumumab; pts., patients; w, week; mRS, modified Rankin Scale; CASE, clinical assessment scale for autoimmune encephalitis; MMSE, mini-mental state examination; MoCA, Montreal cAssessment; NPI, neuropsychiatric inventory.

The study was approved firstly by the Institutional Review Board of the Third Affiliated Hospital of Soochow University. The first patient was randomized in Jan 2025. The participants will be randomly assigned to receive one of three treatment arms: B-cell-depleting agents (ofatumumab followed by daratumumab, B-cell-depleting agents (ofatumumab alone), or repeated IVIG/PE therapy. The flowchart outlining the recruitment, intervention, and evaluation processes is presented in Figure 1.

### 2.2 Study population

#### 2.2.1 Inclusion criteria

The patient inclusion criteria were as follows:

- Aged 12 years and older.
- Diagnosis of autoimmune encephalitis targeting neural surface antigen, predominantly NMDARE.
- At least 3 days of high-dose methylprednisolone pulse therapy (500–1000 mg) and IVIG (0.4 g/kg/day for 5 consecutive days), at least 5 sessions of plasma exchange or immunoadsorption, or at least 2 doses of FcRn antagonist efgartigimod have been used.
- mRS score ≥3 and neuropsychiatric manifestations inadequate to symptomatic treatment.
- Provision of informed consent by the participant or legal guardian.
- Negative pregnancy test for women of childbearing potential (up to 2 years postmenopause).

After providing informed consent, participants will enter a 14-day screening period. For individuals unable to provide consent owing to the severity of their condition, a legally authorized representative (or an equivalent individual as specified by local law) may provide informed consent, ensuring compliance with local regulations.

#### 2.2.2 Exclusion criteria

The exclusion criterion was as follows:

- The investigator determined that the participant had a severe active or chronic infection.
- Participation in another clinical study involving investigational treatment within 4 weeks or 5 half-lives of any previously reported investigational therapy (whichever is longer) prior to randomization.
- Women who are pregnant, breastfeeding, or planning to become pregnant at any time within 6 months after the last dose of the study drug.
- A known history of allergies or reactions to any component of the study drug formulation or a history of allergic reactions following any biological therapy.
- Screening tests confirming positive serology for hepatitis B (HBsAg and/or anti-HBc positive) and/or positive PCR for hepatitis C.
- A history of cancer, except for ovarian teratomas (also known as dermoid cysts), germ cell tumours or nonmelanoma skin cancers (such as squamous cell carcinoma or basal cell carcinoma). Treatment for squamous cell carcinoma and basal cell carcinoma must have been successfully completed with documented cure at least 3 months prior to randomization.
- Administration of any live or attenuated vaccines within 3 weeks prior to enrollment (inactivated vaccines may be administered).
- Administration of the Bacillus Calmette–Guérin (BCG) vaccine within the past year prior to enrollment.

### 2.3 Randomization and treatment allocation

Randomization is performed using a dedicated and password-protected clinical trial platform, Data Acquisition Platform–Electronic Data Capture system (DAP-EDC). Eligible patients are assigned in a 2:2:1 ratio to the three treatment groups. Stratified block randomization was applied, with study center as the stratum. Subjects were allocated sequentially according to their enrollment time.

### 2.4 Informed consent procedure

Before participating in the clinical trial procedures, participant’s guardian will voluntarily complete and sign an informed consent form. The consent form will be paper-based, and a licensed physician will explain its contents, giving the participant sufficient time to make an informed decision. The consent of the research participants must be obtained in accordance with the ethical principles and standards outlined in the Declaration of Helsinki. All consent forms used will be those approved by the IRB, and a copy of the signed consent form will be provided to the participant. If the consent form is amended, the changes will be explained, and the consent process will be repeated.

For participants aged 12–18 years, written informed consent will be obtained from the participant’s legal representative, and written assent will also be obtained from the adolescent participant whenever appropriate, in accordance with institutional ethics requirements for pediatric research (21).

For participants who are unable to provide informed consent due to impaired consciousness or severe neuropsychiatric symptoms related to NMDARE, written informed consent will be obtained from a legally authorized representative (such as a parent, spouse, or other legally recognized representative) in accordance with institutional ethical requirements. Documentation verifying the legal relationship between the representative and the participant will be required. If the participant later regains decision-making capacity, the study procedures will be explained again and written informed consent will be obtained directly from the participant whenever possible.

### 2.5 Study intervention

The RADIA trial includes a 14-day screening period used to determine participant eligibility. Prior to this screening period (specifically, within 90 days before randomization), individuals must have received first-line treatments such as high-dose intravenous corticosteroids, intravenous immunoglobulin (IVIg), and/or plasmapheresis. Importantly, participants who have received at least 2 doses of FcRn antagonist efgartigimod are still eligible for enrollment. The treatment period of the RADIA trial begins at the time of randomization. Participants will be randomized to receive either BCDT followed by daratumumab, BCDT, or repeated IVIG/PE.

The strategy of BCDT refers to multiple sclerosis. Anti-CD20 ofatumumab is administered subcutaneously at a dose of 20 mg at weeks 0, 1, and 2 (loading doses), followed by 20 mg every 4 weeks from week 4 to week 24 (maintenance doses: weeks 4, 8, 12, 16, 20, 24).

Daratumumab will be administered intravenously on the day after each ofatumumab administration. The daratumumab dosing schedule is: 8 mg/kg at weeks 0, 1, 2, and 4 (loading dose), followed by 4 mg/kg at weeks 8, 12, 16, 20, and 24 (maintenance dose). Pre-medication (corticosteroids, antihistamines, and antipyretics) is required before each daratumumab infusion to minimize infusion-related reactions.

In this study, each administration of daratumumab is defined as one treatment cycle. A total of 9 administrations are planned at weeks 0, 1, 2, 4, 8, 12, 16, 20, and 24, resulting in a total treatment duration of approximately 24 weeks. The effects of this daratumumab regimen will be evaluated throughout the study period. Laboratory tests will be required before each dose of the study drug to determine if any dose adjustments are necessary. During the study, patients should receive comprehensive supportive care as appropriate, aligned with their institutional guidelines. Necessary concomitant medications or treatments to ensure adequate supportive care, such as anti-epileptic drugs, may be prescribed. The following concomitant medications are strictly prohibited during the study: natalizumab, cyclosporine, mitoxantrone, mycophenolate mofetil, and azathioprine.

For adolescent patients aged 12–18 years, the doses of study drugs are consistent with the dosing regimen for adult patients. The safety and efficacy of ofatumumab in this age group have been established, and the dose is identical to that used in adults (22–25).

### 2.6 Study measurements and data collection

Disease severity will be evaluated via mRS and CASE, See Table 1 for the specific schedule of assessments for each visit.

**Table 1:** Schedule of enrolment, interventions, and assessments.

	Study period																			
	Screen	Treatment phase													Follow-up					
Time point (week)	−1	0	1	2	3	4	5	6	7	8	12	16	20	24	28	32	36	40	44	48
Enrolment																				
Informed consent	**×**																			
Demographics	**×**																			
Medical history	**×**																			
Inclusion/exclusion	**×**																			
Intervention																				
Ofatumumab		**×**	**×**	**×**		**×**				**×**	**×**	**×**	**×**	**×**	Depends on the level of memory B cells monitored					
Daratumumab		**×**	**×**	**×**		**×**				**×**	**×**	**×**	**×**	**×**						
Rituximab		**×**												**×**	Depends on the level of memory B cells monitored					
IVIG/PE						**×**				**×**	**×**									
Assessments	×																			
HCG test (for female participants)	**×**																			
Physical examination	**×**	**×**	**×**	**×**	**×**	**×**	**×**	**×**	**×**	**×**	**×**	**×**	**×**	**×**	**×**	**×**	**×**	**×**	**×**	
Clinical blood tests and biochemistry	**×**		**×**	**×**		**×**				**×**	**×**	**×**	**×**	**×**			**×**			**×**
Cerebrospinal fluid (CSF) Analysis	**×**																			
Serum immunoglobulin and anti-NMDAR antibody levels	**×**					**×**				**×**	**×**	**×**	**×**	**×**			**×**			**×**
CSF anti-NMDAR antibody levels)	**×**																			
Lymphocyte Subsets	**×**		**×**	**×**		**×**				**×**	**×**	**×**	**×**	**×**			**×**			**×**
Memory B cells and plasma cell subsets	**×**		**×**	**×**		**×**				**×**	**×**	**×**	**×**	**×**			**×**			**×**
mRS	**×**	**×**	**×**	**×**	**×**	**×**	**×**	**×**	**×**	**×**	**×**	**×**	**×**	**×**	**×**	**×**	**×**	**×**	**×**	**×**
CASE	**×**	**×**	**×**	**×**	**×**	**×**	**×**	**×**	**×**	**×**	**×**	**×**	**×**	**×**	**×**	**×**	**×**	**×**	**×**	**×**
MMSE	**×**	**×**	**×**	**×**	**×**	**×**	**×**	**×**	**×**	**×**	**×**	**×**	**×**	**×**	**×**	**×**	**×**	**×**	**×**	**×**
MOCA	**×**	**×**	**×**	**×**	**×**	**×**	**×**	**×**	**×**	**×**	**×**	**×**	**×**	**×**	**×**	**×**	**×**	**×**	**×**	**×**
NPI	**×**	**×**	**×**	**×**	**×**	**×**	**×**	**×**	**×**	**×**	**×**	**×**	**×**	**×**	**×**	**×**	**×**	**×**	**×**	**×**
Imaging	**×**													**×**						**×**
Concomitant medications and dosage	**×**	**×**	**×**	**×**	**×**	**×**	**×**	**×**	**×**	**×**	**×**	**×**	**×**	**×**	**×**	**×**	**×**	**×**	**×**	**×**
Statistical analysis												**×**								**×**

Assessments will be conducted by the study team before randomization; after each study drug cycle at weeks 6, 16, and 24; and during follow-up at week 48. Neurocognitive performance will be measured via the Mini-Mental State Examination (MMSE), the Montreal Cognitive Assessment (MoCA), and the Neuropsychiatric Inventory (NPI) will be anti-neuronal antibody titres and other relevant markers will be analysed in serum and cerebrospinal fluid at baseline and week 16 following the initiation of the study drug treatment. For further details, please refer to the enrollment, intervention, and assessment schedule outlined in Table 1. Routine blood parameters will be assessed before each administration of the study drug, on day28 after each cycle, and at week 16 to monitor for potential drug-induced side effects, including thrombocytopenia, leukopenia, and elevated liver enzymes. Additionally, the length of stay in the ICU after the commencement of the study drug will be recorded at the conclusion of the study. Adverse events, including serious adverse events (SAEs), will be monitored continuously from the first administration of the study drug until 1 year after treatment initiation or until withdrawal from the clinical trial, whichever occurs first. Safety monitoring will therefore cover both the treatment period (up to Week 24) and the subsequent follow-up period (Weeks 24–48).

To reduce assessment bias, all outcome evaluations, including mRS, CASE, and neurocognitive assessments are performed by trained neurologists who are not involved in treatment allocation. All assessment sessions are video recorded and subsequently reviewed by at least one additional independent neurologist blinded to treatment group assignment. In cases of discrepancy, a third independent neurologist adjudicates the final score, ensuring that final outcome scores are based on a blinded adjudication process.

Patient safety monitoring: An independent Data Safety Monitoring Board (DSMB) will be established to oversee patient safety throughout the trial. The DSMB will review unblinded safety data at regular intervals, including cumulative serious adverse events (SAEs), adverse events of special interest (e.g., infusion-related reactions, infections, cytopenias), and protocol deviations. Meetings will be convened every 4 weeks during the treatment phase (weeks 0–24) and every 8 weeks during the follow-up phase (weeks 24–48), with *ad hoc* meetings as needed based on safety signals. Adverse events (AEs) and serious adverse events (SAEs) will be assessed and graded by site investigators using the Common Terminology Criteria for Adverse Events (CTCAE) version 5.0. All AEs and SAEs will be recorded in the electronic Case Report Forms (eCRFs) and reported to the DSMB within 24 h of identification. Causality assessments will be performed by the site investigators and independently reviewed by the DSMB. Safety data will be collected at every scheduled visit as outlined in Table 1. The DSMB will provide recommendations to the sponsor regarding trial continuation, modification, or termination based on pre-specified safety stopping criteria. Safety reporting will follow the CONSORT harms guidelines to ensure transparent and comprehensive documentation of all adverse events.

### 2.7 Strategies to improve adherence to interventions

Standardized patient education with clear instructional materials and simplified dosing schedules will be explained in detail to patients or legal representatives. Regular monitoring of adherence through clinic visits and patient-reported diaries; proactive management of treatment-related adverse events to minimize discomfort; and supportive engagement with caregivers will be carried out to reinforce consistent administration. The investigators will maintain regular, empathetic communication to build trust and encourage sustained compliance.

### 2.8 Discontinuation of the study protocol

The study maybe terminated for individual patients if the patient or their legal representative withdraws consent. Additionally, the investigator may terminate a patient’s participation at any time during the study if the patient meets any of the following termination criteria:

- *Hepatic function*: AST/ALT levels are ≥ 3 times the upper limit of normal (ULN), bilirubin levels are ≥ 2 times the ULN (with direct bilirubin accounting for at least 35% of the total), or the INR is > 1.5 (note that INR values do not apply to patients taking anticoagulants).
- *Cardiac safety*: Investigational medicinal products (IMPs) must be discontinued in patients who meet one of the following ECG criteria on the basis of the average of three readings: QTcF >500 ms, uncorrected QT > 600 ms, or QTcF change from baseline > 60 ms.
- *Infection*: The patient developed suspected new latent tuberculosis infection (LTBI), active tuberculosis (TB), or nontuberculous mycobacterial (NTM) infection during the study (including but not limited to confirmed conversion by interferon gamma release assay [IGRA] or other diagnostic methods).
- *Adverse reactions*: Significant unexpected adverse reactions, unexpected reactions of high severity, or other important safety results as determined by the Independent Data Monitoring Committee (IDMC).
- The trial site may also terminate or suspend the trial early if serious violations of good clinical practice (GCPs), the study protocol, or contractual agreements are identified, or if the site is unable to ensure sufficient enrollment of subjects.

The DSMB will regularly review the progress of the study and conduct interim assessments of safety data. They will provide recommendations to the sponsor regarding whether the nature, frequency, and severity of adverse reactions related to the study treatment warrant early termination or modification of the study to maximize patient benefit. If necessary, the sponsor may decide to terminate the trial.

### 2.9 Withdrawal and replacement of individual subjects

Patients can leave the study at any time for any reason if they wish to do so without any consequences. The attending physician can decide to withdraw a subject from the study for medical reasons. After withdrawal, these patients will receive the standard ICU care.

### 2.10 Outcome measures

The primary objective of the RADIA study was to determine whether treatment with BCDT followed by daratumumab would yield a more effective and rapid treatment response than standard immunotherapy, as measured by changes in mRS. The mRS is a well-established tool for assessing the degree of dependence and disability in daily activities (26); a decrease in the mRS score indicates increased independence and autonomy. Only patients with an mRS score ≥ 3 were included in the RADIA study, as this score indicates severe disability, where the patient is unable to walk independently and requires assistance with activities of daily living. The primary endpoint of the study was the proportion of patients who achieved an mRS score of ≤ 2 at 16 weeks following treatment initiation.

The secondary objectives of the study were to analyse the effects of daratumumab on disease severity at various time points throughout the study, as well as its impact on clinical assessment scales (e.g., Clinical Assessment Scale for Autoimmune Encephalitis (CASE) scores, MoCA, MMSE, NPI and laboratory parameters (e.g., blood immunoglobulin, lymphocyte subsets, memory B cells, and plasma cell subsets). Specifically, the secondary endpoints will evaluate the effects of daratumumab on the following:

- mRS score at 6, 16, 24, and 48 weeks following the first dose of the drug.
- CASE scores at 6, 16, 24, and 48 weeks following the first dose of the drug.
- Duration of ICU if patients are staying in ICU at treatment initiation.
- Cognitive function assessments (MoCA, MMSE, and NPI) will be performed at baseline and at each scheduled study visit, including infusion visits, with the primary analysis conducted at Week 16.
- Absolute and relative changes in serum autoantibody are measured at baseline and 16 weeks after the first dose of the study drug. To ensure methodological consistency across centers, all antibody testing will be conducted by a third-party central laboratory (KingMed Diagnostics, Guangzhou, China) using standardized commercial cell-based assays (CBA). Antibody positivity and titer interpretation will be determined according to the diagnostic cut-off values and procedures recommended by the assay manufacturers and established clinical protocols for autoimmune encephalitis. Specifically, serum and CSF antibody titers are determined by serial twofold dilutions. Positivity is defined as a serum titer ≥ 1:10 or a CSF titer ≥ 1:1, with a clear cell-surface fluorescence intensity of ≥ 2 + (on a 0–4 + scale) compared to the negative control, in accordance with the manufacturer’s instructions. Borderline results (e.g., titer = 1:10 with 1 + intensity) will be retested in duplicate; persistently borderline results will be reported as indeterminate.
- Immune status, including lymphocyte subsets, memory B cells, and plasma cell subsets.
- Safety parameters, including serious adverse events, were monitored within 16 weeks after the first dose of the study drug.

### 2.11 Sample size calculation

The sample size was calculated based on the primary endpoint, defined as the proportion of patients achieving a modified Rankin Scale (mRS) score ≤ 2 at 16 weeks after treatment initiation. Due to the absence of robust published data from randomized controlled trials in this specific patient population, the initial response rate assumptions were derived from limited retrospective studies and clinical experience in patients with severe autoimmune encephalitis.

Under the primary assumption of a 90% response rate in the BCDT followed by daratumumab group and a 70% response rate in the BCDT alone group, with a two-sided significance level of 0.05 (*α* = 0.05) and 80% power (*β* = 0.20), the estimated required sample size was 64 patients per group. After accounting for a 10% dropout rate, approximately 80 participants were estimated for each of the two active comparison groups. For the third group (repeated IVIG/PE), given the anticipated difficulty in recruitment and its role as an exploratory reference arm, the sample size was set at 40 patients. Thus, the total planned sample size was 200 patients.

### 2.12 Statistical analysis

#### 2.12.1 Definition of the primary efficacy endpoint and event definitions

The primary efficacy endpoint is the proportion of patients with an mRS score ≤ 2, assessed at 16 weeks after treatment initiation.

Relapse event: Defined as the occurrence of new or worsening neurological or psychiatric symptoms attributable to NMDARE following an initial period of at least 2 months of clinical improvement or stabilization, accompanied by at least one of the following: (1) recurrence or an increase in anti-NMDAR antibodies in cerebrospinal fluid (CSF) or serum; (2) new or worsening abnormalities on brain MRI consistent with encephalitis; or (3) new or increased epileptiform activity on EEG. All relapse events must be reviewed and confirmed by an independent adjudication committee (6, 21, 27, 28).

Rescue treatment event: Defined as the initiation of any additional immunotherapy beyond the assigned study treatment due to insufficient clinical improvement, clinical deterioration, or life-threatening complications. This includes, but is not limited to, cyclophosphamide, bortezomib, tocilizumab. The decision to initiate salvage therapy is made by the treating physician and subsequently reviewed and confirmed by the independent adjudication committee (21, 29).

#### 2.12.2 Analysis methods for primary and secondary endpoints

For the primary endpoint, the comparison of the proportion of patients achieving mRS ≤ 2 at week 16 between treatment groups will be performed using a Cochran–Mantel–Haenszel test stratified by study center, with a two-sided significance level of 0.05. A logistic regression model adjusting for stratification factors and baseline covariates (e.g., age, baseline mRS) will be used as a supportive analysis.

For time-to-event endpoints, including relapse and salvage treatment events, Kaplan–Meier curves will be used to summarize event times, and event rates with 25%/75% quartiles will be calculated. The stratified log-rank test will be used for intergroup comparisons, and hazard ratios with 95% confidence intervals will be estimated using a Cox proportional hazards model, with stratification factors included as covariates.

#### 2.12.3 Methods for additional analyses

If the degree of censoring is high, a restricted mean survival time analysis will be performed as a sensitivity analysis to support the main estimator. For missing data in the primary endpoint, multiple imputation under the missing-at-random assumption will be applied, and sensitivity analyses will be conducted under alternative missing-data assumptions to assess the robustness of the primary outcome. In addition, sensitivity analyses will be performed under different response rate assumptions to evaluate the stability of the results.

#### 2.12.4 Analysis populations

The Intent-to-Treat (ITT) population was defined as all randomized patients who received at least one dose of the study drug. The Full Analysis Set (FAS) population was defined as patients who met the inclusion criteria and completed at least one efficacy assessment. The primary analysis will be performed on the ITT population. If the difference in key baseline characteristics or primary outcome between the FAS and ITT populations exceeds 5%, the primary analysis will additionally be performed on the FAS population.

For patients who withdraw from the study due to severe adverse events or other reasons, data after withdrawal will be handled using last observation carried forward (LOCF) in sensitivity analyses. For time-to-event analyses, such patients will be censored at the time of withdrawal under the non-informative censoring assumption.

### 2.13 Data management

Source documents are all original records of clinical findings, observations, or other activities used to evaluate and replicate clinical trials. Examples include, but are not limited to: medical records, clinical findings, cranial MRIs for evaluation of intracranial lesions, participant diaries for recording treatment compliance, and patient-reported outcomes (mRS, CASE, MMSE, and MoCA) collected via electronic devices.

Data collection and recording are performed by the investigator under the supervision of the principal investigator, and electronic case report forms (CRFs) are the primary method of data collection. Investigators must ensure the accuracy, completeness, readability, and timeliness of the data contained in case report forms and other records. Data recorded in CRFs must be consistent with source documents; any discrepancies or omissions must be accompanied by explanations. Changes to electronic case report forms must be tracked through an audit trail system.

To ensure data quality, investigators and clinical research coordinators (CRCs) at each study site received training on the study design and eCRF data entry prior to study initiation, ensuring uniform clinical data standards. During eCRF development, internal logic checks were embedded to minimize data entry errors. Data managers reviewed the data twice weekly; when data quality issues were identified, queries were generated within the system, and CRCs verified and corrected the data against source documents, thereby ensuring the quality of clinical data collection.

Trained staff at each study site entered data into an electronic case report form (eCRF). Data were collected via web-based electronic data capture (EDC) software, which automatically logged any changes made to the data. Each patient dataset was assigned a unique identification number upon entry into the study database. Access to individualized patient data was restricted to specific study sites, monitoring personnel, and authorized third parties, including auditors and inspectors.

### 2.14 Ethical approval

Ethical approval was obtained from the leading Ethics Committee at The Third Affiliated Hospital of Soochow University (F-IRB-SOP-00710). As this is a multicenter study, all participating centers have completed the required local ethical review or ethical filing procedures before initiating patient enrollment.

## 3 Summary

Autoimmune encephalitis is a severe and potentially life-threatening condition associated with significant risks of disability. The recovery process for patients with severe manifestations can be prolonged and fraught with complications. While the number of patients accurately diagnosed with this condition is increasing, there are currently no approved pharmacological therapies and no data from randomized controlled trials to inform treatment. Consequently, treatment options are predominantly based on empiric opinion and clinical experience. First-line immunotherapies (steroids, plasma exchange and/or IVIG) with or without second-line immunotherapies (e.g., cyclophosphamide and rituximab) indicate that patients achieve ‘good’ outcomes (mRS score ≤2) in approximately 70% of patients with NMDARE, the most common subtype of AE. Nevertheless, a well-recognized problematic cohort that fails to improve with these interventions and requires ICU admission remains. This unmet need has prompted a search for additional therapies with different mechanisms of action. Owing to the severity of neurological deficits and substantial mortality observed in severe NMDARE, aggressive immune suppression targeting different aspects of humoral immunity is warranted. Sequential therapy consisting of BCDT (rituximab or ofatumumab) followed by daratumumab may provide a stronger suppression of humoral immunity by targeting both B cells and antibody-secreting plasma cells. This study is the first to investigate the efficacy of treatment with B-cell-depleting agents followed by daratumumab in achieving plasma cell depletion, reducing pathogenic antibody titres, and ultimately improving outcomes for patients with severe autoimmune encephalitis. The primary endpoints of the study is the proportion of patients with an mRS score ≤2. Although the mRS does not capture the diverse clinical manifestations of autoimmune encephalitis comprehensively, it has become the most widely utilized clinical outcome measure in stroke trials and has been applied to evaluate autoim mune encephalitis outcomes. Furthermore, through structured assessments, we aimed to collect data on memory function deficits and neuropsychological status related to the disease and explore the potential beneficial effects of daratumumab on these symp toms. By addressing these multifaceted aspects of the condition, this study contributes valuable insights into the management and treatment of AE.

By addressing these multifaceted aspects of the condition, this study contributes valuable insights into the management and treatment of AE.

This trial is conducted solely in China, which may limit the generalizability of findings to other regions with different ethnic backgrounds, healthcare systems, or treatment protocols. Future studies should aim to include a more diverse population to enhance external validity.

Additionally, the open-label design may introduce performance and detection bias, particularly in the assessment of subjective outcomes. To minimize this limitation, several strategies have been implemented. Outcome assessors are independent from the treatment team and blinded to treatment allocation. In addition, all outcome assessments are video recorded and reviewed by an additional independent neurologist, with discrepancies resolved by a third adjudicator, thereby enhancing inter-rater reliability and objectivity. A standardized assessment protocol is applied across all study sites to ensure consistency. Furthermore, the primary endpoint (mRS ≤ 2) is a well-established functional scale with relatively high objectivity.
